# K^+^‐independent Kir blockade by external Cs^+^ and Ba^2+^

**DOI:** 10.14814/phy2.15200

**Published:** 2022-03-11

**Authors:** Ouanounou Gilles

**Affiliations:** ^1^ Université Paris‐Saclay CNRS Institut des Neurosciences Paris‐Saclay Saclay France

**Keywords:** barium, blocking foreign cations, cesium, K^+^ inward rectifier current, mass action law and dissociation‐constant

## Abstract

Cations such as Cs^+^ and Ba^2+^ are known to block K^+^ currents by entering an open channel and binding to the selectivity filter, where they obstruct the pore and block diffusion of the permeant ion. This obstruction is voltage‐ and K^+^‐dependent and is relieved by the *trans* permeant ion flux. The present patch‐clamp study on *Xenopus* muscle cells shows that, unlike the voltage‐activated K^+^ (Kv) channels, blockade of the inward rectifier K^+^ (Kir) channels by external foreign cations results from the combination of pore obstruction with a new and independent mechanism. This new blockade is independent of the K^+^ concentrations and flux and acts indiscriminately on both the outward and the inward Kir components. External Cs^+^ and Ba^2+^ compete for this blockade with free access to common channel sites. These features suggest that the blocking cations do not need to enter the channel for this new mechanism, and should bind to the extracellular side of the channel. When K^+^ fluxes are flowing outward, the pore obstruction is relieved for both Kir and Kv currents, and the K^+^‐independent blockade here described is responsible for a selective Kir inhibition, justifying the use of these external cations as tools in cell physiology studies.

## Graphical abstract

This work evidences a novel mechanism of blockade of the K+‐inward rectifier (Kir) channels by external Cs^+^ and Ba^2+^. This effect is alone responsible for the blockade of the outward component of the Kir currents. In physiological conditions the Kir currents are outwardly directed, and the present work details for the first time the conditions of use of external Cs^+^ and Ba^2+^ for a selective Kir blockade.

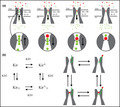

## 1 INTRODUCTION

“K^+^‐inward rectifying” (Kir) channels provide the cell membrane with a constitutively active K^+^ permeability, which decreases if the membrane potential depolarises, and increases if it hyperpolarizes. In excitable cells such as neurons and muscle cells, they participate in the resting potential, in the membrane input conductance, and consequently in the control of excitability (De Boer et al., 2010; Neusch et al., 2003; Zangerl‐Plessl et al., 2019). Inward rectification allows fine‐tuning of the subthreshold membrane conductance, without interfering with the action potential when the threshold is crossed. The study of the physiological roles of an ion channel and of the related electrical conductance often requires the use of a specific blocker. However, few molecules are known to specifically interfere with members of the Kir family (Walsh, 2020; Weaver & Denton, 2021), and nonspecific non‐permeant blocking cations such as Cs^+^ or Ba^2+^ are instead commonly used. The known effects of these cations are not specific, since they were shown to interact with the diffusion process in the selectivity filter, which is common among K^+^ channels such as Kir(s) and Kv(s). Nevertheless, added to the external medium, these blocking cations are empirically used to selectively block the Kir channels. Here I investigated whether and how this practice can indeed lead to a selective blockade.

The theoretical basis supporting the mechanism currently thought to account for the blocking effect of Cs^+^ or Ba^2+^ cations on K^+^ currents was first established by Hille & Schwarz, 1978 (Hille & Schwarz, 1978). This mechanism of the blockade is inherent to a wider model describing the ion diffusion process through K^+^ channels. From this biophysical point of view, K^+^ channels are considered as multi‐ion pores, such that several permeant ions may be present within the selectivity filter of the channel at the same time. Ions move in a single file, interacting with both the channel and each other during the diffusion process (Hille & Schwarz, 1978). This diffusion is not free, and the Eyring rate theory applied to multi‐ion pores was the only model able to describe the dependency of the K^+^ flux on the K^+^ concentrations. According to this model, the channel is considered in terms of a free‐energy profile, describing the pattern of ion free‐energy as a function of its position along the channel. Energy barriers delimit sites of preferential occupancy and formalize the interaction between the permeant ion and the channel. Multi‐occupancy of the selectivity filter of a K^+^ channel by the permeant ion was validated by structural studies on the bacterial KcsA K^+^ channel (Zhou & MacKinnon, 2003). In terms of the multi‐ion pore model, the blocking ion enters the channel pore and takes place in the ions queue, crosses some energy barriers but bumps onto an impassable one, thus interrupting the diffusion process (Hille & Schwarz, 1978) (Figure 7a). This mechanism of the blockade, called here the "obstruction" mechanism, is responsible for specific blockade features. First, the necessity for the blocking cation to cross a fraction of the electrical field before reaching the impassable barrier gives an exponential voltage‐dependency to the blockade, dependent on the blocking ion driving force. Second, as accession to the blocking site by the foreign ion depends on its insertion into the permeant ion file and bumping against an impassable energy barrier, the blocking effect results of a “valve” effect and depends on the K^+^ flux, and consequently on the K^+^ concentrations: For blockade of the inward component of a K^+^ current by an external blocking ion, an increase in internal K^+^ concentration reduces the blockade, whereas an increase in external K^+^ concentration enhances it (Ciani et al., 1980; Hille & Schwarz, 1978; Senyk, 1986). Third, and consequently to the “valve” effect, the blockade is relieved by *trans* permeant ion flux (Hille & Schwarz, 1978) (Figure 7a). Thereby, the effects of internal foreign cations are restricted to the outward component of K^+^ currents, while those of external foreign cations are restricted to the inward component of K^+^ currents. This property was verified on Kv currents in frog myelinated axons (Dubois & Bergman, 1975), where the outward currents were sensitive to internal Cs^+^ only, and the inward tail currents to the external Cs^+^ only. Concerning Kir currents, an extensive literature describes the selectivity‐filter obstruction by external Cs^+^ (Gay & Stanfield, 1977; Hagiwara et al., 1976) and Ba^2+^ (Hagiwara et al., 1978; Standen & Stanfield, 1978) on the inward Kir component. Application of the Eyring rate theory established that Ba^2+^ exercises a mono‐ion block of the selectivity filter, while Cs^+^ can exercise a multi‐ion block, explaining the cooperativity and the strong voltage‐dependence of its effect (Ciani et al., 1980; Hille & Schwarz, 1978; Senyk, 1986). Multi‐occupancy of the KcsA K^+^ channel by Cs^+^ and one‐ion block exercised by Ba^2+^ were finally confirmed by crystallography studies (Jiang & MacKinnon, 2000; Zhou & MacKinnon, 2003). Surprisingly, contrary to Kv channels, there is no information in this abundant literature about the effect of external blocking cations on the outward component of Kir currents.

The question the effect of external Cs^+^ or Ba^2+^ on the outward component of Kir currents is crucial since these cations are empirically used as selective Kir channel blockers (recent examples in neurosciences (Amarillo et al., 2018; Cui et al., 2017; Dell’Orco et al., 2017; Li et al., 2019; Méndez‐González et al., 2020; Patterson et al., 2021; Sebastianutto et al., 2017)). In normal physiological conditions, the membrane potential is indeed rarely more negative than the K^+^ equilibrium and both Kir and Kv currents are outwardly directed, a situation where the only characterized blockade mechanism, that is, obstruction of the selectivity filter, can theoretically not account for a blockade. In restricted physiological situations, Kir currents can be inwardly directed when K^+^ accumulates in the extracellular space, and only if this accumulation is local and/or if other conductances maintain a membrane potential more negative than the depolarized K^+^ equilibrium. However, the use of Cs^+^ and Ba^2+^ commonly goes far beyond these restrictive situations: Most of the studies explore the role of Kir channels in control of the membrane resistance, in a potential range where Kir currents are outwardly directed.

If these cations are empirically used to selectively block Kir channels when added to the extracellular medium, it's because they indeed interfere with the outward component of the related currents, without affecting Kv currents, but the characterization of such an effect remains to be done. In the present voltage‐clamp study, performed on the primary culture of Xenopus myocytes, I took advantage of the large outward component of the muscle constitutively active Kir current and of the fact that in these cultured cells it largely dominates the resting membrane conductance. This work confirms that the K‐dependent obstruction of the selectivity filter restricts its effects to the inward Kir component, but shows that Cs^+^ and Ba^2+^ also bind to external sites and induce a Kir blockade independent of the K^+^ concentrations and on the K^+^ flux direction. Thus this novel mechanism is alone responsible for the blockade of the outward component of Kir current and adds its effects to the obstruction of the selectivity filter when the Kir current is inwardly directed. Characterization of this new mechanism explains for the first time how the use of external Cs^+^ or Ba^2+^ indeed leads to a selective Kir blockade.

## 2 METHODS

### 2.1 Primary cell culture

Myotomal tissue from 1‐day‐old *Xenopus laevis* embryos (stage 22–24) was mechanically dissociated using a Ca^2+^‐ Mg^2+^‐free medium with the following composition (in mM): 115 NaCl, 2.6 KCl, 0.4 EDTA, 10 HEPES, pH = 7.6. The cells were plated on glass coverslips and grown at 20°C for 1–3 days before experiments. The culture medium consisted of 50% Leibovitz L‐15 medium (Gibco, Invitrogen Corp., Cergy‐Pontoise, France), 1% fetal calf serum, 1% antibiotic mixture (*ibid*., final concentration: 100 units//ml penicillin G and 100 µg//ml streptomycin), and 48% physiological solution with the following composition (in mM): 113 NaCl, 2 KCl, 0.7 CaCl_2_, 5 HEPES, pH = 7.8.

### 2.2 Electrophysiology

Whole‐cell patch‐clamp recordings were performed at room temperature (20–22°C). Pipettes were made from borosilicate glass (Clark Electromedical Instruments, Reading, England) and pulled on a P‐87 puller (Sutter Instrument Company, Novato, CA, U.S.A.). Patch electrodes had a resistance of 2.5–3.5 MΩ when filled with internal physiological solution (see below). Membrane currents were recorded using an Axopatch200B patch‐clamp amplifier (Axon Instruments, Union City, CA, U.S.A.), and they were filtered through an integrated 8‐pole low‐pass Bessel filter at 2 kHz. The filtered signals were digitized by a 12‐bit A/D converter (Digidata 1200B, Axon Instruments.) and were stored using pCLAMP 8 software (Axon Instruments). Data were acquired at a sample rate of 10 kHz. Recordings were analyzed using Origin 7 software (OriginLab Corp., Northampton, MA, U.S.A.), taking advantage of the built‐in regression functions. Routines for manipulating data were developed by G.O. using the integrated “Labtalk” programming language.

The standard external and internal solutions had the following compositions (in mM): 140 NaCl, 3 KCl, 2 CaCl_2_, 1 MgCl_2_, 10 HEPES, pH = 7.4 (external solution) and 110 KCl, 10 NaCl, 2 MgCl_2_, 2 EGTA, 10 HEPES, pH = 7.2 (internal solution). The internal solution was filtered through 0.2 µm Millex filters (Millipore, Saint‐Quentin en Yvelines, France). The osmotic pressures of the internal and external solutions were 280–290 mOsm and 300–305 mOsm, respectively, as measured with a freezing‐point osmometer (Knauer, Berlin, Germany). The external medium surrounding the recorded cell could be exchanged within <1 s using a laboratory‐made fast solution changer. BaCl_2_ and CsCl (Normatom grade) were from PROLABO‐VWR (Fontenay‐sous‐Bois, France) and RbCl (*pro analysis* grade) from Merck‐VWR. ML‐133 hydrochloride was from TOCRIS (Bristol, UK).

### 2.3 Membrane currents analysis

For each cell, membrane currents were successively recorded in absence of blocking cations, in presence of non‐saturating concentrations of Cs^+^ or Ba^2+^, and in presence of a 20 mM saturating concentration of Cs^+^. For each cell, membrane currents recorded in presence of 20 mM Cs^+^ were subtracted from the control membrane currents and from the membrane currents recorded in presence of intermediate Cs^+^ or Ba^2+^ concentrations. This operation subtracts pipette leak, membrane leak, capacitive, and all other Cs^+^ resistant currents, among which K(v) currents. For each cell, Kir currents remaining in presence of intermediate Cs^+^ or Ba^2+^ concentrations were divided by the control Kir current, resulting in “fractional currents” (fraction of unblocked current). Fractional currents were averaged over cells and plotted against the membrane potential. Averaged fractional currents were measured at different membrane potentials and plotted against the blocking cation concentration. Fractional currents plotted against the blocking cation concentration were fitted with the Langmuir–Hill equation in order to determine the dissociation constants.

### 2.4 Equations

The following equations were all derived from the mass‐action law.

Equation 1: Langmuir–Hill equation. This equation expresses the fractional currents (relative current remaining in presence of a blocker) as a function of the blocker concentration and of the dissociation constant.

Consider the following equilibrium (Equilibrium 1) between the unblocked Kir channel and the blocked Kir‐L channel (which bound the blocking ligand L):

$$\text{Kir}+L\overset{\text{KD}}{⇆}{\text{Kir}}_{‐L}$$

By definition, the dissociation constant KD is: $\text{KD}=\frac{\left[\text{Kir}\right]\times \left[L\right]}{\left[{\text{Kir}}_{‐L}\right]}$ In practice, as concentrations of channel populations are not accessible quantities, it is usual to calculate the fractional currents, equal to the fraction of unblocked channels: IL/I0 where IL is the current remaining in presence of L and I0 the current in absence of L.

In terms of channel “concentrations”: $\frac{\text{IL}}{I0}=\frac{\left[\text{Kir}\right]}{\left[\text{Kir}\right]+\left[{\text{Kir}}_{‐L}\right]}$.

Given the definition of the dissociation constant, the fractional currents can be expressed as follow:

(1) $$\frac{\text{IL}}{I0}=\frac{\frac{\left[{\text{Kir}}_{‐L}\right]\times \text{KD}}{\left[L\right]}}{\frac{\left[{\text{Kir}}_{‐L}\right]\times \text{KD}}{\left[L\right]}+\left[{\text{Kir}}_{‐L}\right]}=\frac{1}{1+\frac{\left[L\right]}{\text{KD}}}$$

Equation 2: This equation expresses the apparent dissociation constant (KDapp) for the ligand L2 in presence of the ligand L1, in a situation of direct competition (see Equilibrium below). With currents here instead of radio‐labeled ligands, this equation is similar to Cheng & Prusoff, 1973 (Cheng & Prusoff, 1973).

Consider Equilibrium 1 between the unblocked Kir and the blocked Kir−L1 channel states in presence of the L1 ligand:

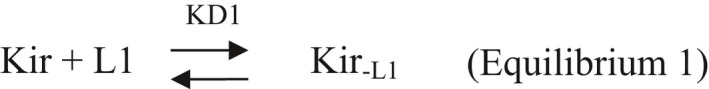

Consider now the Equilibrium 2 between the unblocked Kir′, the blocked Kir′−L1, and the blocked Kir′−L2 channel states in presence of the L1 and L2 ligands:

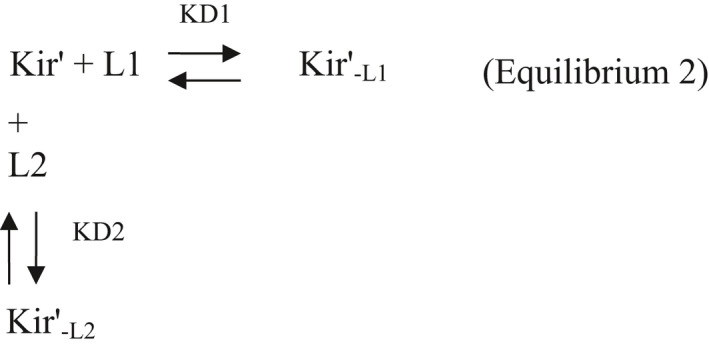

Then:

(a) $$\begin{array}{c} {\text{K}}_{\text{D}\text{1}}=\frac{\left[\text{Kir}\right]\times \left[\text{L}1\right]}{{\text{Kir}}_{‐L1}}=\frac{\left[{\text{Kir}}^{\prime }\right]\times \left[\text{L}1\right]}{{\text{Kir}}_{‐L1}^{\prime }} \end{array}$$

(b) $$K_{\text{D}\text{2}}=\frac{\left[{\text{Kir}}^{\prime }\right]\times \left[L2\right]}{\left[{\text{Kir}}_{‐L2}^{\prime }\right]}$$

(c) $$\begin{array}{c} \left[\text{Kir}\right]+\left[{\text{Kir}}_{‐L1}\right]=\left[{\text{Kir}}^{\prime }\right]+\left[{\text{Kir}}_{‐L1}^{\prime }\right]+\left[{\text{Kir}}_{‐L2}^{\prime }\right] \end{array}$$

(d) $$\begin{array}{c} \left[\mathrm{Kir}\right]=\frac{\left[{Kir}^{\prime }\right]+\left[Kir_{‐L1}^{\prime }\right]+\left[Kir_{‐L2}^{\prime }\right]}{1+\left[\frac{L1}{K_{D1}}\right]} \end{array}$$

deduced from (a) and (c).

Fractional currents remaining in the presence of L1 and L2, relative to L1 alone:

$$\begin{array}{cc} \frac{I_{L1L2}}{I_{L1}}=\frac{\left[Kir^{\prime }\right]}{\left[\mathrm{Kir}\right]} & =\frac{\left[Kir^{\prime }\right]}{\frac{\left[Kir^{\prime }\right]+\left[Kir_{‐L1}^{\prime }\right]+\left[Kir_{‐L2}^{\prime }\right]}{1+\frac{\text{[L}1]}{K_{D1}}}}\\ & =\frac{1+\frac{\text{[L}1]}{K_{D1}}}{1+\frac{\text{[L}1]}{K_{D1}}+\frac{\text{[L2}]}{K_{D2}}}\\ & =\frac{1}{1+\frac{\text{[L2}]}{K_{D2}+K_{D2}\times \frac{\text{[L}1]}{K_{D1}}}} \end{array}$$

Therefore,

(2) $$K_{\text{Dapp}}=K_{D2}+K_{D2}\times \frac{\left[L1\right]}{K_{D1}}$$

Equation 3: This equation expresses the apparent dissociation constant of a ligand L which can bind two independent channel sites with different affinities, leading to independent blockades.

Consider the Equilibrium 3 between the unblocked Kir, the blocked Kir_‐L_, the blocked Kir^−L^ and the blocked Kir_‐L_^−L^ channel states:

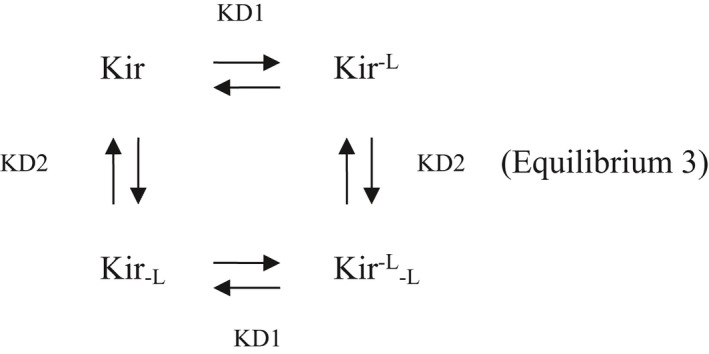

Then:

(f) $$K_{D1}=\frac{\left[\mathrm{Kir}\right]\times \left[L\right]}{\left[Kir^{‐L}\right]}=\frac{\left[Kir_{‐L}\right]\times \left[L\right]}{\left[Kir_{‐L}^{‐L}\right]}$$

(g) $$K_{D2}=\frac{\left[\mathrm{Kir}\right]\times \left[L\right]}{\left[Kir_{‐L}\right]}=\frac{\left[Kir^{‐L}\right]\times \left[L\right]}{\left[Kir_{‐L}^{‐L}\right]}$$

(h) $$\frac{\left[Kir_{‐L}^{‐L}\right]}{\left[Kir\right]}=\frac{\left[Kir^{‐L}\right]}{\left[Kir\right]}\times \frac{\left[Kir_{‐L}\right]}{\left[Kir\right]}=\frac{L}{K_{D1}}\times \frac{L}{K_{D2}}$$

deduced from (f) and (g)

Fractional current remaining after L application:

$\begin{array}{c} \begin{array}{c} \frac{I_{L}}{I_{0}}=\frac{\left[\mathrm{Kir}\right]}{\left[\mathrm{Kir}\right]+\left[Kir^{‐L}\right]+\left[Kir_{‐L}\right]+\left[Kir_{‐L}^{‐L}\right]}\\ \;=\frac{1}{1+\frac{\left[L\right]}{K_{D1}}+\frac{\left[L\right]}{K_{D2}}+\frac{\left[L\right]}{K_{D1}}\times \frac{\left[L\right]}{K_{D2}}}=\frac{1}{1+\frac{\left[L\right]}{\frac{K_{D1}\times K_{D2}}{\left[L\right]+K_{D1}+K_{D2}}}} \end{array} \end{array}$

Therefore,

(3) $$K_{\text{Dapp}}=\frac{K_{D1}\cdot K_{D2}}{\left[L\right]+K_{D1}+K_{D2}}$$

Equation 4: In the context previously described (see Equilibrium 3), that is. one channel with two independent binding sites for the same ligand, this equation expresses the theoretical fractional currents I_L_′/I_0_ which should be obtained in the absence of the blockade mechanism 1. In other terms and for our experiments, this equation expresses the fractional currents corrected from the effects of the L binding to the K^+^‐independent channel sites.

$$\begin{array}{c} \begin{array}{c} \frac{I_{L}^{\prime }}{I_{0}}=\frac{\left[\mathrm{Kir}\right]+\left[Kir^{‐L}\right]}{\left[\mathrm{Kir}\right]+\left[Kir^{‐L}\right]+\left[Kir_{‐L}\right]+\left[Kir_{‐L}^{‐L}\right]}\\ \;=\frac{\left[\mathrm{Kir}\right]}{\left[\mathrm{Kir}\right]+\left[Kir^{‐L}\right]+\left[Kir_{‐L}\right]+\left[Kir_{‐L}^{‐L}\right]}\\ \;+\frac{\left[\mathrm{Kir}\right]}{\left[\mathrm{Kir}\right]+\left[Kir^{‐L}\right]+\left[Kir_{‐L}\right]+\left[Kir_{‐L}^{‐L}\right]}\times \frac{\left[Kir^{‐L}\right]}{\left[\mathrm{Kir}\right]} \end{array} \end{array}$$

Therefore,

(4) $$\frac{I_{L}^{\prime }}{I_{0}}=\frac{I_{L}}{I_{0}}+\frac{I_{L}}{I_{0}}\times \frac{\left[L\right]}{K_{D1}}$$

## 3 RESULTS

### 3.1 External Cs^+^ blocks both inward and outward components of the Kir current, but only the inward component of the Kv currents

**FIGURE 1:**
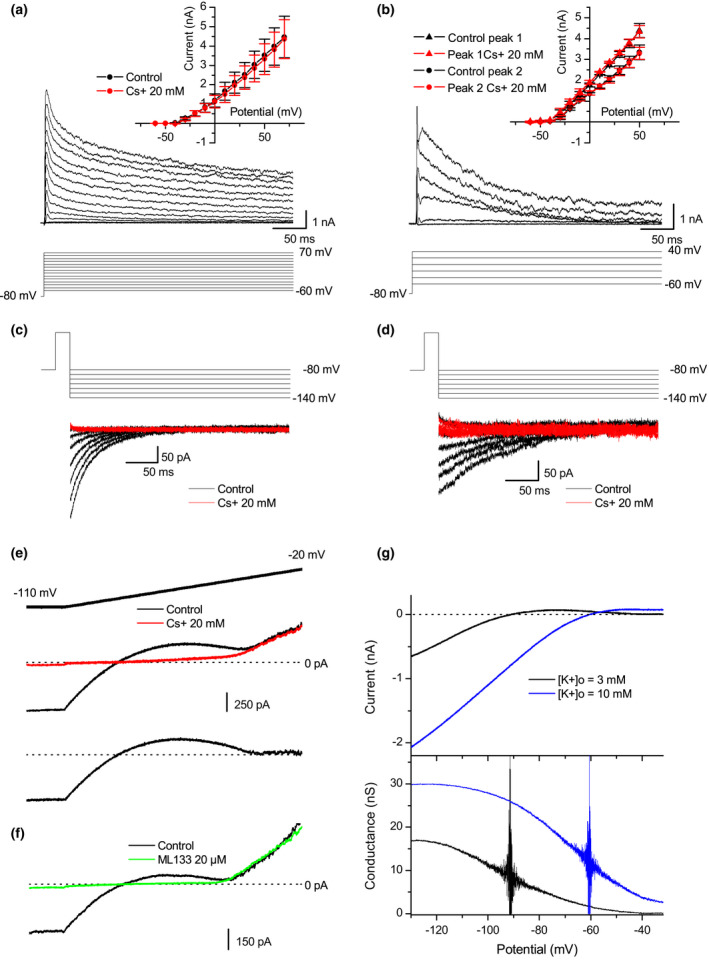
External Cs^+^ blocks both the inward and outward components of the Kir current, but only the inward component of the Kv currents. (a) TEA‐sensitive outward K^+^ currents activated by a classical 10 mV step voltage‐clamp protocol. Traces are shown after the subtraction from the control recordings of the currents in the presence of TEA 20 mM. *Inset*: Mean TEA‐sensitive outward peak currents plotted against the membrane potential, in the absence (black circles) and in the presence (red circles) of external Cs^+^20 mM (mean ± SEM, *n* = 4). (b) 4AP‐sensitive K^+^ currents, obtained as in (a) by subtraction from the control recordings of the currents in the presence of 4AP 3 mM. *Inset*: Mean 4AP‐sensitive K^+^ outward peak currents plotted against the membrane potential, in the absence (black symbols) and in the presence (red symbols) of external Cs^+^20 mM (mean ± SEM, *n* = 3). Triangles and circles refer to the two peaks visible on each trace (see text). (C) TEA‐sensitive “tail‐currents” were recorded after a 80 mV, 20 ms activating step in the absence (black traces) and in the presence (red traces) of external Cs^+^20 mM. (d) 4AP‐sensitive “tail‐currents” were recorded after a 80 mV, 20 ms activating step, in the absence (black traces) and in the presence (red traces) of external Cs^+^20 mM. (e) Membrane currents were recorded in standard solutions (E_K_ = −90 mV, middle trace) during a voltage‐clamp ramp protocol (−110 mV–0 mV, 150 mV/s, top trace), in the absence (black) and in the presence (red) of Cs^+^20 mM. The difference between the two traces (i.e., the Kir current blocked by Cs^+^) is shown on the bottom trace. (f) Membrane currents were recorded in standard solution during a voltage ramp protocol, in the absence of ML133 (black trace) and 8 min after 20 µM ML133 application (green trace). (g) Kir currents (top panel) recorded during a negative ramp potential (−125 mV/s) in [K^+^]_o_ = 3 mM (black trace) and in [K^+^]_o_ = 10 mM (blue trace). The corresponding chord membrane conductance is shown in the bottom panel. Vertical lines are due to the high noise‐to‐current ratio around the K^+^ equilibrium potential

Subtraction of the membrane currents recorded in the presence of a blocker from those recorded in its absence gives the currents sensitive to the blocker. Using a common voltage step protocol and external tetraethylammonium (TEA), this operation delineated a transient outward rectifying Kv current on *Xenopus* myocytes (Figure 1a, *n* = 4). The transient behavior of the *Xenopus* TEA‐sensitive current is like that found for skeletal muscle cells (Nakajima et al., 1962), and contrasts with the TEA‐sensitive Kv currents of the myelinated axon (Dubois, 1983), which are more slowly activated and inactivated. In the presence of external 4‐aminopyridine (4‐AP), similar protocols and analyses gave outward rectifying Kv currents composed of a fast transient current and a slower one (Figure 1b, *n* = 3). This difference in kinetics distinguished two peaks (Figure 1b), having distinct I/V relationships (Figure 1b *inset*). The complex character of the 4‐AP sensitive currents is well known in other cell types, for example, in the axon (Quinta‐Ferreira et al., 1982). As shown in the insets of Figure 1a,b, the mean I/V relationships of these outward K^+^ currents were not significantly affected by the presence of 20 mM Cs^+^ in the external medium. In contrast, the inward tail currents recorded after an activation voltage step were completely abolished by external Cs^+^ (Figure 1c,d). As reported with the frog myelinated axon (Dubois & Bergman, 1975), it is here confirmed that outward K^+^ flux relieves the blockade exercised by external Cs^+^ on the muscle Kv currents, in accordance with the view that blocking ions obstruct the selectivity‐filter of the corresponding channels with a “valve” effect (Figure 7a).

The muscle Kir current was initially described by Katz (1949) and called the "anomalous rectifying K^+^ current." In mammals, this current is now called IK1, and known to be driven through Kir2.1 channels (De Boer et al., 2010; DiFranco et al., 2015; Jurkat‐Rott & Lehmann‐Horn, 2004). Figure 1e shows the membrane currents recorded during a 150 mV/s potential ramp in the absence (black trace) and in the presence of 20 mM external Cs^+^ (red trace). During the ramp in control conditions (black trace), the inward current initially decreased, reversed around –90 mV, became outwards with a bell shape up to –40 mV, then increased nearly linearly. This latter part (from −40 mV up) corresponds to the activation of the Kv currents described above, whereas the first part (from −110 to −40 mV) should represent essentially the Kir current. In order to show that this current is driven through the *Xenopus* ortholog of the mammalian Kir2.1 channel, the selective Kir2.x inhibitor ML 133 was applied at 20 µM (Figure 1f, green trace), a low concentration that fully block Kir2.1 channels (Wang et al., 2011). The full blockade of the inward rectifying component (from −110 to −40 mV) strongly suggests that it is driven through Kir2.1 channels. Very similarly to 20 µM ML 133, in the presence of 20 mM external Cs^+^ (Figure 1e, red trace), the remaining currents reversed between −90 and −70 mV, and did not rectify up to −40 mV, above which they could not be distinguished from the outward Kv currents. Therefore, in contrast to Kv currents, both inward and outward components of the Kir current were blocked by 20 mM external Cs^+^ (same results were obtained with 300 µM Ba^2+^, not shown). The Cs^+^‐resistant currents thus comprise a non‐rectifying and mainly K^+^ leak, Kv outward currents and capacitive currents. Subtraction of the membrane currents recorded in the presence of Cs^+^ from the control recordings thus gave the Cs^+^‐sensitive Kir component, since Kv currents were not flowing inwards (Figure 1e, lower trace). A way to observe the behaviour of the Kir current at large hyperpolarizations is to extend the ramp experiment shown in Figure 1e toward more negative potentials (in practice using a negative voltage ramp to preserve membrane integrity). Such a ramp was used in Figure 1g (upper panel), in the presence of two different external K^+^ concentrations. After subtraction of the Cs^+^‐resistant currents, the resulting I/V relationships show that the reversal potential of the Kir current strictly followed the Nernst equilibrium potential for K^+^ ions, in accordance with the reported strong selectivity of Kir channels. Conversion of these I/V relationships into macroscopic conductances (Figure 1g, lower panel) illustrates the inward rectification process, as shown by the decrease in the Kir conductance to zero at 50 mV above E_K_. As would be expected for a Kir channel (Hagiwara & Yoshii, 1979; Leech & Stanfield, 1981), the absolute Kir conductance strongly depended on the external K^+^ concentration, as well as the rectification process which slides along the membrane potential axis.

Contrary to the *Xenopus* myocyte Kv currents, the outward component of the Kir current therefore appeared sensitive to external Cs^+^ and Ba^2+^. Aside from the particular situation of the protocol used in Figure 1c,d to record inward Kv tail currents, Kv is not flowing inward since they activate above their reversal potential, and the specific sensitivity of the outward Kir component to external foreign cations provides the opportunity for a selective Kir blockade. In the following, for each cell and at the end of each recording session, the membrane currents were recorded in the presence of a saturating Cs^+^ concentration (20 mM). The remaining currents, comprising all other native, pipette‐leak and capacitive currents were thus subtracted from recordings. In the following, current traces are shown after this subtraction was performed.

### 3.2 External Cs^+^ and Ba^2+^ block the outward component of the Kir current independently of K^+^

**FIGURE 2:**
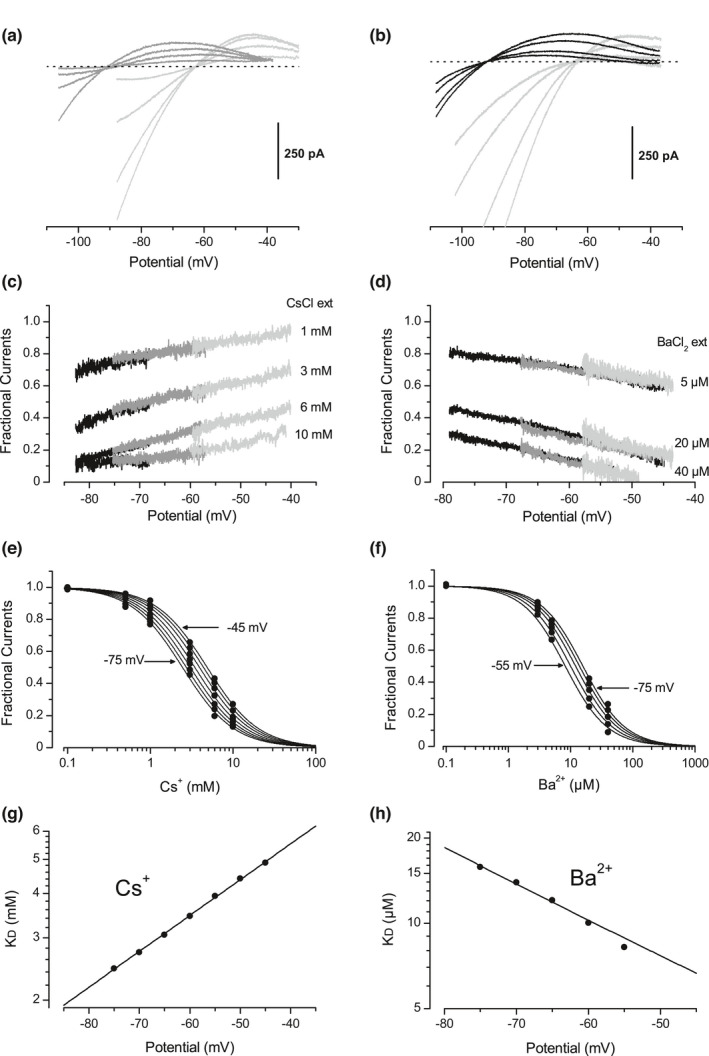
Effect of external Cs^+^ and Ba^2+^ on the outward component of the Kir current. (a) Averaged Kir currents (*n* = 5 for each K^+^ gradient) generated by a voltage‐clamp ramp protocol (150 mV/s) under control conditions and with 1, 3, or 6 mM of external Cs^+^, in different K^+^ concentration gradients: [K^+^]_i_ = 110 /[K^+^]_o_ =3 mM (grey traces), and [K^+^]_i_ = 110/[K^+^]_o_ = 10 mM (light grey traces). (b) Averaged Kir currents (*n* = 5 for each K^+^ gradient) in control conditions and with 5, 20 or 40 µM of external Ba^2+^, in different K^+^ gradients: [K^+^]_i_ = 110/[K^+^]_o_ = 3 mM (black traces), and [K^+^]_i_ = 110/[K^+^]_o_ = 10 mM (light grey traces). (c) Mean fractional outward currents (*n* = 5 for each K^+^ gradient) remaining after Cs^+^ addition, plotted against the membrane potential for three K^+^ concentration gradients: [K^+^]_i_ = 150/[K^+^]_o_ = 3 mM (black traces), [K^+^]_i_ = 110/[K^+^]_o_ = 3 mM (grey traces), and [K^+^]_i_ = 110/[K^+^]_o_ = 10 mM (light grey traces). (d) Mean fractional outward currents (*n* = 5 for each K^+^ gradient) remaining after Ba^2+^ addition, plotted against the membrane potential for three K^+^ concentration gradients: [K^+^]_i_ = 110/[K^+^]_o_ = 3 mM (black traces), [K^+^]_i_ = 110/[K+]_o_ = 10 mM (light grey traces), and [K^+^]_i_ = 170/[K^+^]_o_ = 10 mM (grey traces). (e) Mean fractional currents (circles) plotted against the Cs^+^ concentration, as computed from the data shown in (c) at various membrane potentials. Solid lines are given by Equation (1), with *n* = 1.4. (f) Mean fractional currents (circles) plotted against the Ba^2+^ concentration, as computed from the data shown in (d) at various membrane potentials. Solid lines are given by Equation (1), with *n* = 1.28. (g) Cs^+^ dissociation constant plotted against the membrane potential, derived from the fits presented in (e). The solid line is given by a monoexponential function with a voltage constant of 43 mV. (h) Ba^2+^ dissociation constant plotted against the membrane potential, derived from the fits presented in (f). The solid line is given by a monoexponential function with a voltage constant of −34 mV

The sensitivity of the outward Kir component to external Cs^+^ and Ba^2+^ does not fit with the mechanism of selectivity filter obstruction, and suggests that there is no interaction between the blocker and the permeant ion. In order to show that, the effects of various concentrations of external Cs^+^ (Figure 2a, *n* = 5 for each K^+^ gradient) and Ba^2+^ (Figure 2b, *n* = 5 for each K^+^ gradient) on the outward component of the Kir current have been thus quantified in different K^+^ concentration gradients. Fractional Kir currents (fractions of the control Kir current remaining after blocker addition) were calculated for each cell and then averaged, giving the mean unblocked fraction of the outward Kir component as a function of the membrane potential (Figure 2c,d, for Cs+and Ba^2+^, respectively). For each blocker concentration, the fractional currents obtained in different K^+^ gradients superimposed exactly. Therefore, in contrast with the Kir reversal potential and conductance, the blocking effects of external Cs^+^ and Ba^2+^ appeared to be independent of the K^+^ concentrations. To estimate the apparent affinities of these cations for the channel sites responsible for this blocking effect, the fractional currents extracted from the averaged data shown in Figure 2c,d have been plotted against the blocker concentration (Figure 2e,f, respectively). The dissociation constant K_D_ was then determined as a function of the membrane potential, by fitting these data points with Equation (1) (Langmuir‐Hill equation, see Methods), derived from the mass action law:

(1) $$\frac{I_{L}}{I0}\left(V\right)=\frac{1}{1+{\left(\frac{\left[L\right]}{K_{D}\left(V\right)}\right)}^{n}}$$

where I_0_ and I_L_ are the Kir currents in the absence and in the presence of the blocking ligand L, K_D_ the dissociation constant, and n the Hill coefficient, respectively. The best fits were obtained with n set to 1.45 for Cs^+^ and 1.28 for Ba^2+^. The resulting K_D_ values are plotted against the potential in Figure 2g,h, and could be fitted to a mono‐exponential function for both Cs^+^ and Ba^2+^ but with opposite potential constants (43 and −34 mV, respectively). A Hill coefficient comprised between 1 and 2 means that for each blocking ion, more than one channel binding site is involved in the blockade with a partial positive cooperativity. In this case, the K_D_ value given by Equation (1) corresponds to an “average” K_D_, as the nth root of the product of all of the individual K_D_ values.

Blockade of the outward component of the Kir current by external Cs^+^ and Ba^2+^ therefore appeared independent of the K^+^ concentrations, further supporting the idea that this effect is not mediated by an obstruction of the Kir selectivity filter. This K^+^‐independent effects was voltage dependent for both blocking cations, but with opposite voltage constants.

### 3.3 Cs^+^ and Ba^2+^ compete together for blockade of the outward Kir component

**FIGURE 3:**
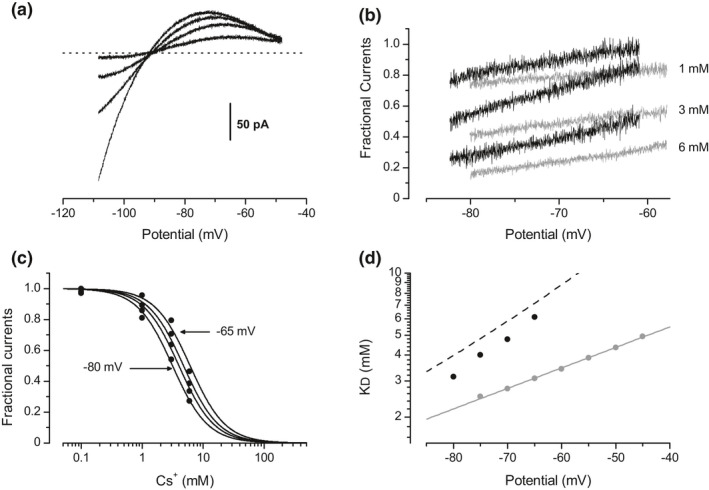
Cs^+^ competes with Ba^2+^ in a blockade of the outward Kir current. (a) Averaged Kir currents (*n* = 6) recorded during a ramp potentials (150 mV/s) in external medium containing 15 µM of Ba^2+^ ([K^+^]_o_ = 3/[K^+^]_i_ = 110 mM), in the absence and in the presence of 1, 3 and 6 mM of Cs^+^. (b) Mean fractional outward currents (*n* = 6) remaining after addition of Cs^+^ in the absence (grey traces, same data as in Figure 2c) or in the presence of Ba^2+^ 15 µM. (black traces, relative to the Ba^2+^ alone condition). (c) Fractional currents, extracted from (b) at various membrane potentials (circles), plotted against the Cs^+^ concentration. The solid lines are fits to Equation (1), with *n* = 1.4. (d) Cs^+^ apparent dissociation constants computed from the fitted curves shown in (c), in the presence of Ba^2+^ (black symbols) and in control conditions (grey symbols, same data as in Figure 2g). The dashed line represents the theoretical apparent affinity given by Equation (2)

Similarities between the Cs^+^ and the Ba^2+^ blocking effects on the outward Kir component raise the question of whether they act with the same mechanism and by binding to common channel sites. Hence, competition experiments were undertaken. Figure 3a shows the mean outward Kir currents (*n* = 6) in an external medium containing 15 µM of Ba^2+^, in the absence or in the presence of three different external Cs^+^ concentrations. Fractional currents remaining after Cs^+^ addition were calculated relative to the Ba^2+^ alone condition (Figure 3b, black traces). Competition was revealed by the shift of the fractional currents toward enhanced values in the presence of external Ba^2+^. Fits of Equation (1) to the fractional currents (Figure 3c) extracted from data shown in Figure 3b resulted in the apparent K_D_ values for the Cs^+^ blocking effect in the presence of 15 µM of external Ba^2+^ (Figure 3d). In the simple case of a single binding site, with free access to this site and a direct competition between Cs^+^ and Ba^2+^ (see Methods, Equilibrium 2), the theoretical K_D_ constant related to the apparent Cs^+^ effect in the presence of Ba^2+^ can be defined by Equation (2), derived from the mass action law (see Methods):

(2) $$K_{D}Csapp=K_{D}Cs+K_{D}Cs.\frac{\left[Ba\right]}{K_{D}Ba}$$

Using the K_D(v)_ relationships related to Cs^+^ (Figure 2g) and to Ba^2+^ (Figure 2h), Equation (2) returned for 15 µM Ba^2+^ a theoretical apparent Cs^+^ affinity function drawn with a dashed line in Figure 3d. This theoretical relationship was close to that experimentally obtained, suggesting a direct competition between Cs^+^ and Ba^2+^ and a free access to common binding sites for mediation of the K^+^‐independent blockade of the outward Kir component.

### 3.4 Selectivity‐filter obstruction and K^+^‐independent blockade add together when Kir current is inwardly directed

Given the K^+^‐independence of the blockade observed here on the outward Kir component, it could be hypothesized that this blockade also operates on the inward Kir component. On the other hand, the selectivity‐filter obstruction by foreign cations theoretically restricts its effects to the inward Kir component. Therefore, study of the inward Kir component was expected to reveal that blockade by external foreign cations results from a combination of both blockade mechanisms (Figure 7b).

**FIGURE 4:**
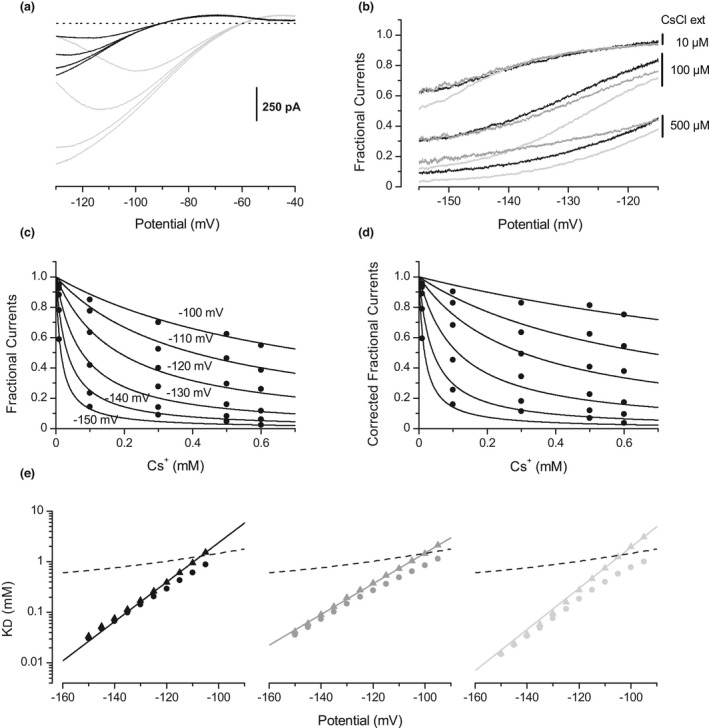
Effect of external Cs^+^on the inward component of the Kir current. (a) Averaged Kir currents (*n* = 6 for each K^+^ gradient) generated by a ramp potentials (−125 mV/s) under control conditions and with 10, 100, and 500 µM of external Cs^+^, in different K^+^ concentration gradients: [K^+^]_i_ = 110/[K^+^]_o_ = 3 mM (black traces) and [K^+^]_i_ = 110/[K^+^]_o_ = 10 mM (light grey traces) (b) Mean fractional inward currents (*n* = 6 for each K^+^ gradient) plotted against the membrane potential, in three K^+^ gradients: [K^+^]_i_ = 110/[K^+^]_o_ = 3 mM (black traces), [K^+^]_i_ = 150/[K^+^]_o_ = 10 mM (grey traces), and [K^+^]_i_ = 110/[K^+^]_o_ = 10 mM (light grey traces). (c) Mean fractional currents in [K^+^]_i_ = 110/[K^+^]_o_ = 10 mM, plotted against the Cs^+^ concentration as derived from the fractional currents presented in (b) at various membrane potentials; solid lines are fits to Equation (1), with *n* = 1. (d) Fractional currents resulting from computation by Equation (4) of data shown in (c). This operation subtracts from the fractional currents the part due to the K^+^‐independent blockade described in Figure 2. The solid lines are fits to Equation (1), with *n* = 1. (e) Cs^+^ dissociation constants plotted against the membrane potential, obtained in the same three K^+^ gradients as in (b) (same grey tones). Circles correspond to the global K_D_(v) relationships for Cs^+^ computed from fits to Equation (1) as shown in (c). Triangles represent the K_D_(v) relationships calculated from fits as shown in (d), after subtraction from the global blockade of the K^+^‐independent component. The solid lines show the fits of Equation (5) to the data, with δ set to 2.27, 2.37 and 1.77 in [K^+^]_I_ = 110/[K^+^]_o_ = 3 mM, [K^+^]_i_ = 110/[K^+^]_o_ = 10 mM, and [K^+^]_i_ = 150/[K^+^]_o_ = 10 mM respectively. The dashed lines show extrapolation of the K^+^‐independent K_D_(v) relationship established for Cs^+^ on the outward Kir current (same data as in Figure 2g)

**FIGURE 5:**
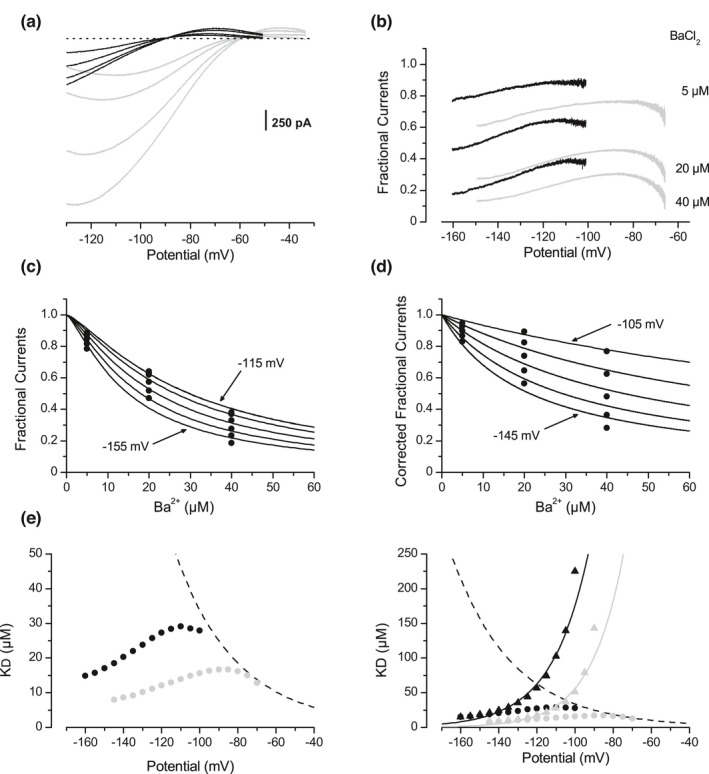
Effect of external Ba^2+^ on the inward component of the Kir current. (a) Averaged Kir currents (*n* = 5 for each K^+^ gradient) recorded during a ramp potentials (−125 mV/s) in the absence and with 5, 20 and 40 µM of external Ba^2+^, in two different K^+^ gradients: [K^+^]_i_ = 110/[K^+^]_o_ = 3 mM (black traces), and [K^+^]_i_ = 110/[K^+^]_o_ = 10 mM (light grey traces). (b) Mean fractional inward currents (*n* = 5 for each K^+^ gradient) plotted against the membrane potential, in the presence of the same Ba^2+^ and K^+^ concentrations as in (a). (c) Mean fractional inward currents plotted against the Ba^2+^ concentration, in [K^+^]_i_ = 110/[K^+^]_o_ = 3 mM. The solid lines are fits to Equation (1), with *n* = 1.3. (d) Fractional currents computed from data shown in (c) using Equation (4). The solid lines are fits to Equation (1), with *n* = 1. (e) Ba^2+^ dissociation constants plotted against the membrane potential. Circle symbols represent the apparent K_D_ values related to the inward Kir blockade, calculated from fits of Equation (1) as shown in (c). Triangle symbols correspond to the K_D_ values computed from fits of fractional currents as presented in (d), after subtraction of the K^+^‐independent blockade. The solid lines are fits of Equation (5) with δ = 0.65 in [K^+^]_i_ = 110/[K^+^]_o_ = 3 mM, and 0.75 in [K^+^]_i_ = 110/[K^+^]_o_ = 10 mM. The dashed lines show extrapolation of the K^+^‐independent K_D_(v) relationship established for Ba^2+^ on the outward Kir current (same data as in Figure 2h)

The effects of various concentrations of external Cs+ (Figure 4a, *n* = 6 for each K^+^ gradient) and Ba^2+^ (Figure 5a, *n* = 5 for each K+ grandient) were quantified in different K+ concentration gradients, focussing on the inward component of the Kir current. The low Cs^+^ concentrations that were able to block the inward Kir component, only slightly affected its outward component. In contrast, the range of Ba^2+^ concentrations necessary to produce a gradual blockade was the same for the inward as for the outward component (same concentrations as in Figure 2d). For both Cs^+^ (Figure 4b) and Ba^2+^ (Figure 5b), fractional inward currents were calculated for each cell and averaged, giving the mean unblocked fraction of the inward component of the Kir current as a function of the membrane potential. In the case of Cs^+^, reversion of the Kir current from outward to inward direction marked a discontinuity, with an increased voltage dependency of the blockade. In the case of Ba^2+^, the fractional inward currents were first in line with those established from the outward Kir component, but curved and reversed their voltage‐dependency with hyperpolarization. For both Cs^+^ and Ba^2+^, and in contrast with fractional outward currents, fractional inward currents were dependent on the K^+^ concentrations. For each K^+^ gradient, the fractional currents expressed as a function of the blocker concentration (see one example in Figure 4c for Cs^+^ and Figure 5c for Ba^2+^) were extracted from the mean fractional currents plotted against the potential in Figures 4b and 5b. The dissociation constant K_D_ was then determined as a function of the membrane potential, by fitting these data points with Equation (1). Best fits were obtained using a Hill coefficient of 1 for Cs^+^ and 1.3 for Ba^2+^, and gave for each K^+^ concentration gradient (distinguished by grey scale) the apparent K_D_ values plotted with circle symbols in Figure 4e for Cs^+^ and Figure 5e for Ba^2+^. Sensitivity to the K^+^ concentrations qualitatively fitted with obstruction of the selectivity‐filter for both Cs^+^ and Ba^2+^: as an example, increase in the external K^+^ concentration favouring inward K^+^ fluxes enhanced efficacy of the blockade. In contrast, the K_D_(v) relationships did not strictly follow the exponential pattern expected for selectivity‐filter obstruction. Deviation from this theoretical pattern was more pronounced for Ba^2+^ than for Cs^+^ but in both cases, the increase in KD with depolarization seemed limited by the KD(v) established on the outward Kir component (Figures 4e and 5e, dashed line).

A possible explanation for these observations is that the K^+^‐independent blockade previously described adds its effects to the selectivity‐filter obstruction when the Kir current is inwardly directed (Figure 7b). Indeed, in a blocking scheme with a single blocking ion species and two independent channel sites mediating independent blockades (see Methods Equilibrium 3 and Figure 7b), the apparent affinity (represented by the apparent dissociation constant K_Dapp_) is related to the elementary dissociation constants K_D1_ and K_D2_ and to the blocking compound concentration [L] by Equation (3), derived from the mass action law (see Methods):

(3) $$K_{\text{Dapp}}=\frac{K_{D1}.K_{D2}}{K_{D1}+K_{D2}+\left[L\right]}$$

The site having the highest affinity, therefore, dominates the global effect. In practice, since K_Dapp_ is estimated from fitted data points where [L] varies, the K_D1_ and K_D2_ cannot be directly extracted with Equation (3) from the apparent inward K_D_(v) relationships. This operation can be made at the level of the fractional currents. Therefore, Equation (4) was derived (see Methods) to subtract the participation of one of the blocking mechanisms from the global fractional inward currents. Assuming independence between the two mechanisms, Equation (4) expresses the fractional currents due to the mechanism 2, as the global fractional currents plus the relative blockade produced by the mechanism 1 (see Methods Equilibrium 3 and Figure 7b):

(4) $${\frac{\mathrm{IL}}{I0}}^{\prime }=\frac{\mathrm{IL}}{I0}+\frac{\mathrm{IL}}{I0}\cdot {\left(\frac{\left[L\right]}{K_{D1}}\right)}^{n}$$

In the present case, ${\frac{\mathrm{IL}}{I0}}^{\prime }$ is the theoretical (fractional currents/[L]) relationship related to the "obstruction" mechanism, $\frac{\mathrm{IL}}{I0}$ is the global fractional current, K_D1_(v) is the K^+^‐independent dissociation constant established from the outward Kir component, and *n* the Hill coefficient. When applied to Cs^+^ and Ba^2+^, Equation (4) gave fractional currents such as those exemplified in Figures 4d and 5d, respectively. The new K_D_(v) functions (Figure 4e for Cs^+^ and Figure 5e for Ba^2+^, triangle symbols) were calculated for each K^+^ gradient by fitting these latter fractional currents with Equation (1). Importantly, for both Cs^+^ and Ba^2+^, and for each K^+^ gradient, subtraction of the K^+^‐independent component from the global blockade resulted in new K_D_(v) functions with an exponential pattern. Corrected K_D_ values could now be fitted with Equation (5), derived from the Woodhull's blockade model (Woodhull, 1973) and commonly used to quantify the voltage‐dependency of the Kir selectivity‐filter obstruction.

(5) $$K_{D}\left(V\right)=K_{DO}.\exp\left(\frac{z\delta VF}{\mathrm{RT}}\right)$$

where K_D0_ is the dissociation constant at 0 mV, δ the electrical distance, V the membrane potential, T the absolute temperature, F and R are the Faraday and the gas constants respectively. Good fits were obtained with fractional field δ from 1.77 to 2.37 for Cs^+^, and from 0.65 to 0.74 for Ba^2+^, in agreement with what has been previously reported in the literature (Alagem et al., 2001; Gay & Stanfield, 1977; Hagiwara et al., 1976, 1978; Murata et al., 2002; Shieh et al., 1998; Standen & Stanfield, 1978).

While blockade of the outward Kir component was due to the K^+^‐independent mechanism only, blockade of the inward Kir component seemed to combine the K^+^‐independent blockade with the obstruction of the selectivity filter. The accuracy with which Equation 4 allows to subtract the K^+^‐independent effect from the global blockade suggested that the two mechanisms are independent of each other.

### 3.5 Selectivity‐filter obstructionand K^+^‐independent blockade act independently of each other

In the previous section, experiments where K^+^ concentrations varied showed that the efficacy of the selectivity‐filter obstruction can be modulated independently of the K^+^‐independent blockade. This observation suggests that these blockade mechanisms are independent of each other (Figure 7b). This feature should be observable in competition experiments with different blocking ions. It should be noted that the obstruction model assumes that an indirect competition occurs between blocking ions. Even if their blocking sites differ inside the selectivity filter, ions move in a queue and depend on each other to reach their own binding sites (see Introduction, Figure 7a).

**FIGURE 6:**
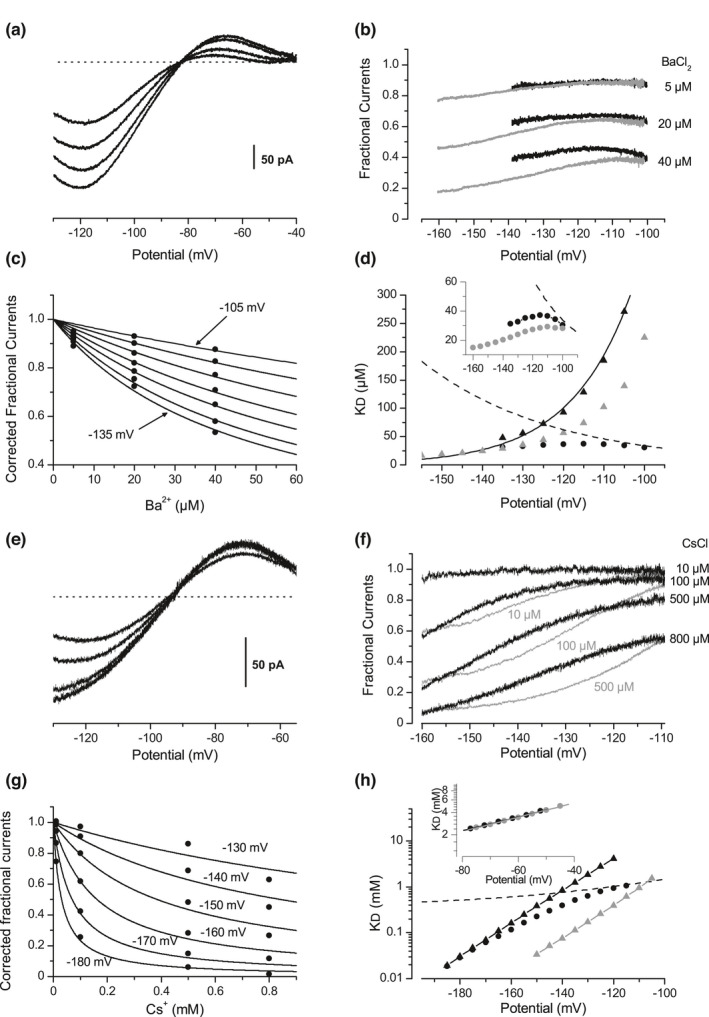
Competition in a blockade of the inward Kir currents. (a) Averaged Kir currents (*n* = 6) recorded during ramp potentials (−125 mV/s) in external medium containing 100 µM of Cs^+^ ([K^+^]_o_ =3/[K^+^]_i_ = 110 mM), in the absence and in the presence of 5, 20 and 40 µM of Ba^2+^. (b) Mean fractional inward currents (*n* = 6) remaining after addition of Ba^2+^ in presence of Cs^+^100 µM (black traces, relative to the Cs^+^ alone condition). Fractional currents in absence of Cs^+^ are shown in grey (same data as in Figure 5b). (c) Fractional inward currents plotted against the Ba^2+^ concentration, after subtraction by Equation (4) of the K^+^‐independent blockade due to Ba^2+^. The solid lines are fits of Equation (1), with *n* = 1. (d) Ba^2+^ dissociation constants plotted against the membrane potential. Black symbols represent the K_D_(v) relationship in the presence of Cs^+^, whereas grey symbols correspond to the control conditions (same data as in Figure 5e). Circles represent the apparent K_D_(v) relationships calculated before the subtraction of the K^+^‐independent blockade. Triangles correspond to the K_D_(v) values calculated after subtraction of the K^+^‐independent blockade, from fits of Equation (1) to data shown in (c). The black line is given by Equation (5) with δ = 0.84. The dashed line is given by a monoexponential function with a potential constant of −34 mV, as an extrapolation of the Ba^2+^ effect on the outward current (same data as in Figure 2h). *Inset* Enlarged view showing the non‐corrected K_D_(v) relationships for Ba^2+^, in the absence (grey symbols) and in the presence (black symbols) of Cs^+^. (e) Averaged Kir currents (*n* = 7) during ramp potentials (−125 mV/s), in the absence and in the presence of 10, 100, 500 or 800 µM of Cs^+^, in an external medium containing 1 mM of Rb^+^ ([K^+^]_o_ = 3 mM/[K^+^]_i_ = 110 mM). (f). Mean fractional inward currents (*n* = 7) after addition of Cs^+^ in the Rb^+^ containing external solutions (black traces, relative to the Rb^+^ alone condition). Fractional currents remaining after Cs^+^ addition in the absence of Rb^+^ are shown in grey (same data as in Figure 4b). (g) Fractional inward currents remaining after Cs^+^ addition in presence of Rb^+^, plotted against the Cs^+^ concentration after subtraction by Equation (4) of the K^+^‐independent blockade due to Cs^+^. The solid lines are fits of Equation (1), with *n* = 1. (h) Black circles represent apparent Cs^+^ dissociation constants in presence of Rb^+^, calculated from fits of Equation (1) before subtraction of the K^+^‐independent blockade. Black triangles represent the Cs^+^ dissociation constants in the presence of Rb^+^, calculated from fits of Equation (1) to the fractional currents corrected from the K^+^‐independent blockade and shown in (g). The Cs^+^ K^+^‐dependent dissociation constants calculated in absence of Rb^+^ and corrected from the K^+^‐independent blockade are shown in grey (same data as in Figure 4e). The dashed line represents the extrapolation of the K^+^‐independent K_D_(v) relationships for Cs^+^ established from blockade of the outward Kir component (same data as in Figure 2g). *Inset* K^+^‐independent K_D_(v) relationships related to the Cs^+^ effect on the outward Kir current, in the absence (grey circles) and in the presence (black circles) of Rb^+^1 mM

The effect of various Ba^2+^ concentrations was studied in the presence of a low Cs^+^ concentration of 100 µM (Figure 6a, *n* = 6). This latter Cs^+^ concentration did not significantly affect the K^+^‐independent blockade exercised by Ba^2+^ on the outward Kir component. Fractional inward currents remaining after Ba^2+^ applications in the presence of Cs^+^ were recalculated relative to the Cs^+^ alone condition (Figure 6b, black traces). Fractional currents were expressed as functions of the Ba^2+^ concentration (data not shown) and fitted with Equation (1), *n* set to 1.3. The resulting apparent K_D_(v) values were plotted in Figure 6d (black circle symbols). Compared to the control condition with Ba^2+^ alone (Figure 6b, grey traces, Figure 6d *inset*, grey circles, same data as in Figure 5b,e, respectively), 100 µM of external Cs^+^ strongly reduced the selectivity‐filter obstruction apparent efficacy, and consequently enhanced the relative participation of the K^+^ independent blockade. The fractional inward currents were then corrected for the K^+^‐independent Ba^2+^ effect using Equation (4), resulting in the new relationships presented in Figure 6c. Fits of these corrected fractional currents with Equation (1), *n* now set to 1, returned the K_D_(v) values graphed with black triangle symbols in Figure 6d. The corrected K_D_(V) functions followed the exponential pattern expected for the selectivity‐filter obstruction.

Rb^+^ is another cation known to block the inward component of Kir currents (Adrian, 1964; Standen & Stanfield, 1980). I checked that external Rb^+^ did not affect the K^+^‐independent blockade exercised by external Cs^+^ on the outward Kir component (Figure 6h, *inset*). Cs^+^ was then applied at various concentrations in the presence of 1 mM Rb^+^ (Figure 6e, *n* = 7). Mean fractional inward currents remaining after Cs^+^ addition were recalculated relative to the Rb^+^ alone condition (Figure 6f, black traces). Fractional currents were expressed as functions of the Cs^+^ concentration (data not shown), and fitted to Equation (1), *n* set to 1. The resulting apparent K_D_(v) values for Cs^+^ in presence of Rb^+^ were plotted with circles symbols in Figure 6h. Using Equation (4), subtraction of the K^+^‐independent Cs^+^ blockade gave the corrected fractional current shown in Figure 6g. Fits to Equation (1) resulted in the corrected K_D_(v) relationship plotted in Figure 6h with black triangle symbols. Once again, the relative contribution of the K^+^‐independent blockade could be enhanced by an apparent decrease in efficacy of the selectivity‐filter obstruction, and subtraction of the K^+^‐independent effect restored the exponential pattern expected for the selectivity‐filter obstruction alone.

In this section, instead of “competition” with the permeant K^+^ cation as in the previous section, competition between blocking cations was used to modulate the degree of blockade by obstruction of the Kir selectivity filter. The subtraction of the K^+^‐independent blockade from the complex blockade of the inward Kir component allowed for each conditions to isolate a pure selectivity filter obstruction, enhancing the view that both blockade mechanisms are independent of each other.

## 4 DISCUSSION

Obstruction of the selectivity‐filter has been the only model considered to account for the effects of Cs^+^ and Ba^2+^ on the K^+^ channels (Hille & Schwarz, 1978), which includes Kv and Kir channels. The present study shows that an additional mechanism of the blockade is responsible for the effects of external Cs^+^ and Ba^2+^ on Kir but not on Kv channels. This new mechanism appeared strictly independent of the K^+^ concentrations and current direction, while as extensively described in previous studies, obstruction of the selectivity‐filter by external foreign cations restricts its effects to the inward component of Kir currents. Therefore, the K^+^‐independent mechanism of the blockade is alone responsible for the blockade of the outward component of the Kir current and adds its effects to the obstruction of the selectivity filter when K^+^ fluxes are inwardly directed. In physiological conditions, the membrane potential is mainly equal or above the K^+^ equilibrium, and the K^+^‐independent blockade being specific to the Kir current, the present study justifies for the first time the use of external Cs^+^ or Ba^2+^ for a selective Kir blockade.

By itself, the sensitivity of the outward component of the Kir current to external Cs^+^ and Ba^2+^ was incompatible with obstruction of the selectivity filter and suggested that another mechanism was responsible for this effect. Moreover, this effect appeared insensitive to changes in the K^+^ concentrations, also suggesting that insertion in the file of permeant ions, as occurs for the selectivity filter obstruction, was not required to mediate this blockade. Direct competition between Cs^+^ and Ba^2+^ (Figure 3) implies free access to common blocking sites and was also incompatible with obstruction of the selectivity filter. Finally, the voltage dependency of the K^+^‐independent blockade exercised by external Ba^2+^ was opposite to that of the Ba^2+^ driving force, suggesting that Ba^2+^ did not need to enter the channel pore to mediate this effect. Concerning external Cs^+^, the sign of the voltage‐dependency of the K‐independent blockade was in agreement with its driving force, but the voltage‐dependency was less marked than expected for a multi‐ions block of the selectivity filter, as reported in other studies (Gay & Stanfield, 1977; Hagiwara et al., 1976) and confirmed here on the inward Kir component (Figure 4). Taken together, these results suggest that external Cs^+^ and Ba^2+^ block the outward Kir component according to a new mechanism, distinct from obstruction of the selectivity filter. This K^+^‐independent blockade is likely mediated by binding of the foreign cations to common channel sites, located before the selectivity filter, possibly on the external side of the channel. This K^+^‐independent mechanism ensures alone the Cs^+^ and Ba^2+^ effects observed in the present study on the outward Kir component.

**FIGURE 7:**
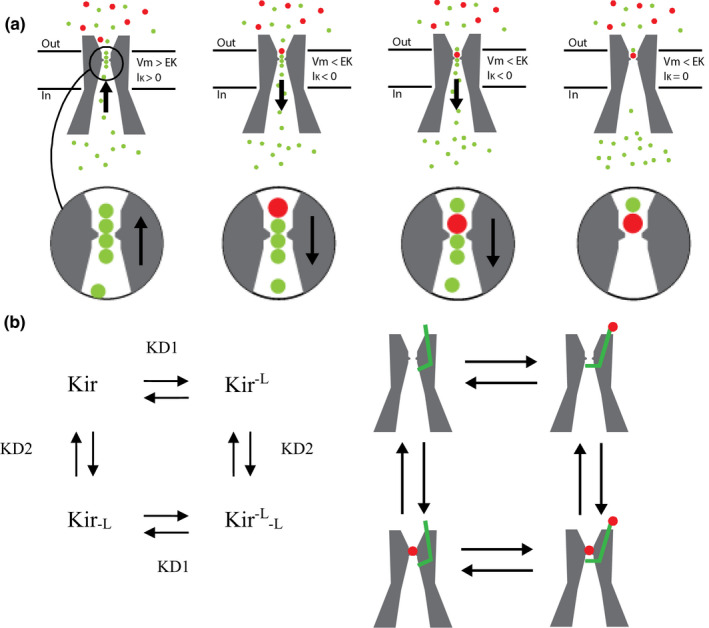
Schematic representation of the dual Kir blockade. (a) Obstruction of the selectivity filter. When K^+^ (green dots) flux is outwardly directed (left panel), external Cs^+^ or Ba^2+^ (red dots) cannot enter the ions file and cannot obstruct the selectivity filter. When K^+^ flux is reversed from outward to inward (subsequent panels), the blocking cation enters the permeant ion queue, but bumps onto an impassable energy barrier and blocks the inward K^+^ current. (b) Schematic representation of the equilibrium between different channel states in the context of the dual blockade, and when K^+^ flux is inwardly directed. Obstruction of the selectivity filter is represented by the blocking cation (red dot) inside the selectivity filter. A K^+^‐independent blockade is represented by a green latch that closes the channel below the selectivity filter when the ligand (red dot) binds an extracellular site

Reversion of the Kir current from outward to inward direction revealed the main features characterizing an obstruction of the Kir selectivity filter by external Cs^+^ and Ba^2+^ (Figures 4 and 5). The blockade was strongly K^+^‐dependent and its voltage‐dependency was in agreement with the involvement of the blocking ion driving‐force. Therefore, it was clear that the well‐known obstruction mechanism was effective on the inward Kir component. However, a detailed analysis of this blockade suggested that the obstruction mechanism did not operate alone. Indeed, the complexity of the blockade was evidenced by the non‐exponential patterns obtained for the apparent K_D_(V) relationships (Figures 4e and 5e). Instead, an increase in the apparent K_D_(V) relationships with depolarization seemed capped by the K^+^‐independent K_D_(V) relationships established from the outward Kir component analysis. Deviation from the theoretical exponential pattern varied in degree with the relative contribution of each mechanism. Thus, when external or internal K^+^ concentrations were decreased or increased respectively, or when competition experiments were performed, decreases in the apparent obstruction efficacy increased the relative contribution of the K^+^‐independent blockade and deviation of the apparent K_D_(V) relationship from its theoretical exponential pattern. Importantly, Equation (4) allowed to subtract the fraction due to the K^+^‐independent mechanism from the global blockade, in normal and competition conditions, and irrespective of the K^+^ concentration. The main argument in favor of the efficacy of this operation was the exponential pattern followed by the “corrected” K_D_(v) relationships. These relationships then could be fitted by the Woodhull Equation (see Figures 4 and 5), resulting in fractional field ẟ concordant with that reported in other studies. The fact that ẟ > 1 for Cs^+^ was in accordance with the strong voltage‐dependency produced by the multi‐ion blockade (Gay & Stanfield, 1977; Hagiwara et al., 1976). Concerning Ba^2+^, sub‐unity ẟ value agreed with a mono‐ion blockade, as reported in other studies (Alagem et al., 2001; Hagiwara et al., 1978; Murata et al., 2002; Shieh et al., 1998; Standen & Stanfield, 1978). Importantly, the fact that the blocking obstruction could be selectively modulated without change in the efficacy of the K^+^‐independent blockade, by modulating the K^+^ concentrations or in competition experiments, shows that these mechanisms are independent of each other, as illustrated in Figure 7b.

The obstruction of the selectivity filter is a passive blocking mechanism, which doesn't require any channel structural change. Although very speculative using electrophysiological data only, it can be hypothesized that the K^+^‐independent blockade revealed here involves a conformational change in the Kir protein structure. Indeed, even if Kir channels are devoid of the S1–S4 helices which provide to the Kv channel their voltage sensitivity (Kim & Nimigean, 2016), x‐ray crystallography and structural studies, essentially performed by Roderick MacKinnon over the two last decades, revealed an intrinsic gating mechanism for Kir channels that could be involved in the K^+^‐independent blockade by foreign cations. In the tetrameric structure of K^+^ channels, each subunit contributes to the pore region, including the selectivity filter, with two transmembranes α helices (TM1 and TM2) connected by a loop (P‐loop). Comparison of the Kir3.x channels (G protein‐gated Kir channel) with constitutively active Kir2.x channels revealed that rotation of the TM2 helix around a “gating hinge,” following binding of the G_i_βγ subunit, leads the Kir3.x channel into an open conformation comparable to the opened Kir2.x channel conformation (Whorton & MacKinnon, 2011, 2013). In addition to this helix gate mechanism, that closes the pore channel at the cytoplasmic side of the pore below the selectivity filter, a second gate system, in series with, and more cytoplasmic than the helix gate, is constituted by a G loop at the apex of the Kir cytoplasmic domain (Pegan et al., 2005). Comparative structural studies of the Kir channel families, in the presence and in the absence of PIP2, G_i_βγ subunit and ATP, finally explained how theses gates coordinate to open the Kir2.x in presence of PIP2 (Tao et al., 2009), how PIP2 and intracellular Na^+^ prepare Kir3.x to be opened by G_i_βγ (Niu et al., 2020; Wang et al., 2016; Whorton & MacKinnon, 2011, 2013), and how Kir6.x are closed by ATP (Lee et al., 2017; Martin et al., 2017). The two interesting points for speculating about the K^+^‐independent blockade mechanism are that conformational changes can lead to a closed channel even for Kir2.x, and that the gating mechanisms close the channel on the cytoplasmic side of the pore, below the selectivity filter. If we hypothesize a binding of Cs^+^ or Ba^2+^ to an extracellular loop linking TM1 to TM2, and that this binding induced a conformational change toward a closed state of the channel (as in the absence of PIP2), the closure below the selectivity filter is not in contradiction with the apparent independence of the K^+^‐independent blockade and the obstruction of the selectivity filter. This independence was symbolized by a gate that closes the channel below the selectivity filter in scheme B of Figure 7. In addition, during the 90’s and the beginning of the century, the cloning, mutations, and expression in heterologous expression systems of several Kir subunits have identified residues involved in the blockade by foreign cations. Interestingly, in addition to the residues found in the P‐region of the channels and presumably participating in the obstruction mechanism (Alagem et al., 2001; Murata et al., 2002; Sabirov et al., 1997; Thompson et al., 2000; Zhou et al., 1996), supplementary channel sites located outside of the selectivity filter (Abrams et al., 1996; Thompson et al., 2000), some of them even external to the pore region (Alagem et al., 2001; Murata et al., 2002), were also found to be involved in the blocking effect of external cations on the inward Kir component. The role of these residues may have to be reexamined in regard of the complexity of the blockade revealed in the present study.

It might seem surprising that the K^+^‐independent blockade described here has not been reported prior to this work. However, it should be noted that Kir currents were generally studied on their inward component, for which the obstruction mechanism predominates in efficacy. Moreover, the blockade of the Kir currents was mainly studied after expression in heterologous systems, in *symmetrical* K^+^ concentrations (about 50 times higher than physiological external concentration). Compared to the present study, the use of supra‐physiological external K^+^ concentrations strongly enhanced the efficacy of the obstruction mechanism and reduced the required concentrations of blocking ions. In these latter conditions, the relative contribution of the K^+^‐independent blockade to the global effect was probably barely visible. In the present work, focus on the outward Kir component and use of physiological K^+^ gradients revealed the K^+^‐independent mechanism and its contribution to the blockade of the inward Kir component.

**FIGURE 8:**
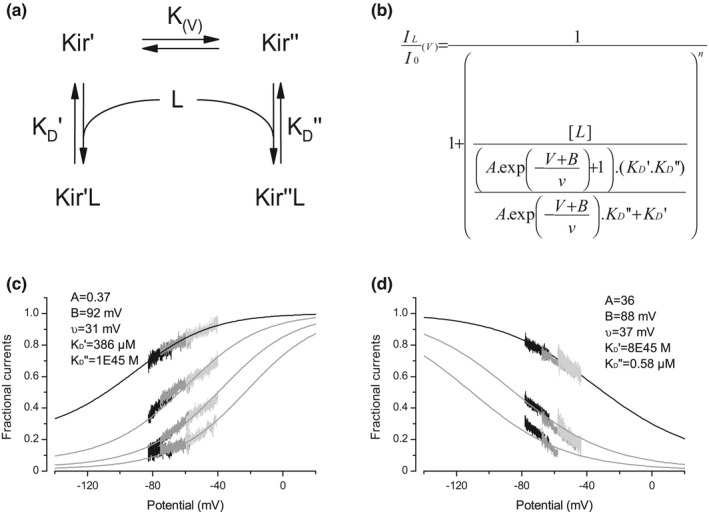
Kir channel functioning may provide the K^+^‐independent effects of external Cs^+^ and Ba^2+^ with a voltage‐dependency. (a) Equilibrium scheme where the ligand L binds two‐channel states Kir′ and Kir′′ linked by a voltage constant K(v), with different affinities KD′ and KD′′ respectively. (b) Equation (6) Theoretical fractional Kir currents remaining in presence of the ligand L, as a function of the L concentration, of the K(v) constant, and of the dissociation constants KD′ and KD′′. (c) Experimental fractional currents remaining in presence of external Cs^+^ shown in Figure 2c. The black line is a fit to Equation (6) performed on the lowest Cs^+^ concentration (1 mM). The K(v), KD′ and KD′′ constants given by the fit were used to calculate the other relationships (grey lines) simply by changing the Cs^+^ are concentration in Equation (6). (d) Experimental fractional currents remaining in presence of external Ba^2+^ shown in Figure 2d. The black line is a fit to Equation (6) performed on the lowest Ba^2+^ concentration (5 µM). The K(v), KD′ and KD′′ constants given by the fit were used to calculate the other relationships (grey lines) simply by changing the Ba^2+^ concentration in Equation (6)

The present results suggest that Cs^+^ and Ba^2+^ exercise their K^+^‐independent effects through the binding to common channel sites, out of the selectivity filter, and that their driving force is not involved in these effects. This raises the question of the origin of the voltage‐dependency of these effects, furthermore opposite for the two foreign cations. A possibility is that the channel itself confers this voltage dependency. Precisely Shieh et al. (1996) proposed an intrinsic voltage‐sensitivity for the Kir2.1 channel with two open states one linked to another by an equilibrium constant dependent on the membrane potential. In such a model with two‐channel states, linked by an equilibrium constant K(v) and with different affinities for the foreign cation, the fractional currents remaining after blocking ion application can be defined by Equation 6:

(6) $$\frac{I_{L}}{I_{0}}=\frac{1}{1+{\left(\frac{\left[L\right]}{\frac{\left(K\left(v\right)+1\right)\left(K_{D}^{\prime }.K_{D}^{\prime \prime }\right)}{K\left(v\right).K_{D}^{\prime \prime }+K_{D}^{\prime }}}\right)}^{n}}$$

where I_0_ and I_L_ are respectively the Kir currents in the absence and in the presence of the blocking ligand L, K_D_′ and K_D_′′ the dissociation constants for Kir′ and Kir,′′ and n the Hill coefficient. When the population of Kir′ decreases with the membrane potential following a Boltzman function, the population of Kir′′ increases with an opposite function, and the ratio K(v) follows an exponential decay function $K\left(V\right)=A.\exp\left(‐\frac{V+B}{v}\right)$. With a K(v) exponential decay function, the fractional currents follow Boltzman functions, the direction of which depends on K_D_′ and K_D_′′. A K_D_′/K_D_′′ ratio less than 1 for Cs^+^ and more than 1 for Ba^2+^ leads to an increasing Boltzman function for fractional currents in the presence of Cs^+^, and to a decreasing one in the presence of Ba^2+^. Fits to Equation 6 have been performed on the fractional currents in the presence of 1 mM of Cs^+^ (Figure 8c, black line) and in the presence of 5 µM of Ba^2+^ (Figure 8d, black line). As expected, these fits returned a low affinity of Cs^+^ for the Kir” state and of Ba^2+^ for the Kir′ state, thus modeling the opposite voltage‐dependencies. Importantly, the grey lines are not model fits. For each foreign cation, the value of the two K_D_s and the K(v) relationship returned by the fit operated on the lowest ligands concentrations were used to calculate the Boltzman fractional currents in the presence of 3, 6, and 10 mM of Cs^+^ (Figure 8c, grey lines), and in the presence of 20 and 40 µM of Ba^2+^ (Figure 8d, grey lines). Despite the simplicity of this model, the fact that these calculated functions match the fractional currents simply by changing the concentrations of the ions strongly support the view that the fractional currents indeed follow Boltzman functions, and that the driving force of the blocking ions, which changes with concentrations, is indeed not involved in these effects.

## CONFLICT OF INTEREST

The author declares that no conflicts of interests exist.

## AUTHOR CONTRIBUTION

G.O. designed the project, performed the experiments and analyses, and wrote the paper.
